# 

**Published:** 1877-07

**Authors:** 


### CONTENTS.

PAGE.

**CONTRIBUTIONS.**

Choose Ye Which. BEN. H. PRATT ..... 253
One Health. One Disease P. H. HAYES, M. D. ..... 255
Probabilities. B. BROWNE ..... 257

**MEDICAL ITEMS AND NEWS.**

Whooping Cough—Pneumonia—Scarlatina—Neuralgic Pill — Chronic Uterine Catarrh — Acute Rheumatism— Diphtheria—Sea-Sickness—Elixir of Pepsin, Bismuth and, Strychnia—Elixir of Beef, Iron and Wine—Hydrobromic Ether as an Anæsthetic—Elixir Calisaya, Iron and Bismuth —Elixir of Pepsin and Bismuth—Anæmia—Palpitation of the Heart—Gonorrhœa—Hoarseness —To stop Bleeding—Prickley Heat—Piles —Belladonna as a Brain Stimulant—Warburg's Tincture—Diphtheric Sore Throat—Elixir of Calisaya, Iron, Bismuth and Strychnia—Cholera Infantum —Expectorant —Artificial Sea-water—Chillblains —Tape-Worm Bolus—New Anæsthetic—Cystitis —Bad Cold Cure—Headache—Inflamed Throats —Iodide of Starch—Uterine Tumors—Tetanus— Strangulated Hernia—Leeches—Citrate of Chinoidine in Fevers—Meningitis—Substitute for Cod-Liver Oil—Digestive Pastilles—Acute Rheumatism and Cold Water—Sunstroke in Children— Preparation of Raw Meat—Myrrh—Choice of Sedatives—Salicylic Acid in Diphtheria—Rheumatism—Feeding of Infants—Spinal Irritation— Burns—Bacteria—Diphtheria ..... 258 to 267

**OUR CORRESPONDENCE.**

Eight Letters with Replies ..... 268

PAGE.

**HOUSEHOLD HINTS AND RECIPES.**

To Clean Varnished Paint—French Lip Salve— Camphor Ice—To Clean White Spar Ornaments —To Remove Freckles—Hair and Scalp Wash— Barber's Shampoo Mixture—Golden Brown Hair Dye—To take Rust out of Steel—Blonde or Flaxen Hair Dye—Bleaching Leaves—New Solvent for Copal—Chronic Acid for Warts—To Clean Soiled Ribbons and Silks—Violet Ink for Rubber Stamps—Cocoanut Soup—To Prevent Glass Breaking—To Remove Ink Marks—To keep Oil Cloths looking well—To Clean Paint — Egg Test—To Stew Celery—Dried Beef—Tar Water as a Dye—Cement for Glass—To Remove Stains from Kids—Alcohol and Cold—Butter and Substitutes—Mock Sweet Breads—"A Reseat fore the Sistum"—Washing Suggestions — Indigestible Substances—Preservation of Food—To Purify a Sick Chamber—Preserving Eggs—To Dry Apples, &c—White Washing—Whitewash for Out-Door Use—Solvent for Rubber ..... 269 to 272

**EDITORIAL.**

FALSE AND STIFF JOINTS ..... 273

ALCOHOLIC ..... 274

FUNERALS ..... 275

CHOLERA INFANTUM ..... 276

HEMIOPIA ..... 276

**EDITORIAL CHIT-CHAT.**

Quick Work—Pansies—Photography—Wild Flowers—The Crops—Aqua Gloriæ—Railway Crossings—Hay Fever—Mr. Towner's Book—Dramatic Talent— Tramps — Jim — Street Grading— Plato's Best Thoughts—City Laborers—Cataract Operations—Our Delay ..... 277 to 280

